# Spontaneous rupture of the urinary bladder following hemorrhoidectomy: A case report

**DOI:** 10.1016/j.ijscr.2025.111790

**Published:** 2025-08-11

**Authors:** Hanieh Raghimi, Rahim Jorjani, Sina Mohajernoei, Nasrollah Abian, Hossein Saffari, Ehsan Zolfi

**Affiliations:** a Department of Medical Science, School of Medicine, Golestan University of Medical Sciences, Gorgan, Iran; b Department of Urology, 5 Azar Hospital, School of Medicine, Golestan University of Medical Sciences, Gorgan, Iran; c Department of Urology, Hasheminejad Kidney Center, School of Medicine, Iran University of Medical Sciences, Tehran, Iran

**Keywords:** Spontaneous rupture of urinary bladder, Hemorrhoidectomy, Urinary retention, Intraperitoneal bladder rupture, Cystography, Case report

## Abstract

**Introduction and importance:**

Spontaneous rupture of the urinary bladder (SRUB) is a rare urological emergency. SRUB often results from over-distension due to acute urinary retention (AUR), which is more common in males but can also occur in females, particularly after surgeries like hemorrhoidectomy. This case highlights SRUB in a woman following hemorrhoidectomy, a rare complication that requires early recognition and management.

**Case presentation:**

A 42-year-old woman presented with abdominal pain and obstructive urinary symptoms after undergoing hemorrhoidectomy. She had no prior abdominal trauma or gastrointestinal symptoms. Initial examination revealed abdominal distention and suprapubic dullness. A Foley catheter was inserted, providing temporary relief. Laboratory findings showed a significant rise in serum creatinine (6.2 mg/dL). CT cystography confirmed an intraperitoneal bladder rupture. The patient underwent open surgery, and her postoperative recovery was uneventful, with a return to normal creatinine levels.

**Clinical discussion:**

SRUB can be associated with anorectal surgeries like hemorrhoidectomy, where iatrogenic nerve injury leads to AUR. The bladder retention can cause an increase in intra-vesical pressure, ultimately resulting in rupture. Early diagnosis is critical, as symptoms may be nonspecific. While ultrasonography provides some clues, CT cystography is the gold standard for diagnosis. Timely surgical intervention for intraperitoneal ruptures prevents life-threatening complications.

**Conclusion:**

SRUB following hemorrhoidectomy is a rare but serious complication. Clinicians should consider SRUB in patients presenting with urinary symptoms, abdominal pain, and elevated creatinine level in the absence of hydronephrosis after pelvic surgeries.

## Highlights

• Spontaneous rupture of the urinary bladder (SRUB) is a rare but possible complication following hemorrhoidectomy.
• Urinary symptoms, pain, and high serum Cr after pelvic surgeries, such as hemorrhoidectomy, should raise suspicion for SRUB.
• Early diagnosis of SRUB is critical to prevent further complications and ensure timely management.
• CT cystography is the gold-standard method for diagnosing SRUB.
• Intraperitoneal SRUB is best treated by surgical repair.

## 1 Introduction

Bladder rupture is an uncommon condition, most often caused by abdominopelvic trauma or as a result of surgical procedures, but it may also occur spontaneously [1]. Spontaneous rupture of urinary bladder (SRUB) is a rare urological emergency which is estimated to occur in 1 out of 126,000 people [2,3]. It is characterized as rupture with no predisposing factor such as trauma, bladder malignancies, neurogenic bladder, and exposure to radiation [4].

The underlying mechanism for SRUB has been suggested to be over-distension of the bladder secondary to acute urinary retention (AUR) with simultaneous weakness in the bladder wall [4]. Acute urinary retention (AUR) is most often seen in males and its occurrence is extremely rare in females considering anatomical differences between the two genders [5]. However, some predisposing factors such as urethral stricture, functional bladder disorder, or pelvic related surgeries may induce AUR even in females. Among various anal surgeries, AUR has been reported most often after hemorrhoidectomy as a postoperative complication [6].

Here, we present a SRUB in a woman after hemorrhoidectomy. We discuss its clinical features, diagnostic challenges, and the management. We also provide literature review in about this subject. This case report has been reported in line with the SCARE criteria [7].

## 2 Case presentation

Our patient was a 42-year-old woman presented to emergency department with abdominal pain and obstructive urinary tract symptoms started from 2 days ago. She did not report any trauma to her abdominopelvic region. Furthermore, there were no gastrointestinal symptoms nor any rectal bleeding. On her past medical history, she was suffering from depression taking sertraline for the last 3 years. 4 days earlier, she had had a hemorrhoidectomy.

Her hemodynamic was stable and body temperature was 37.1 °C. Abdominal examination revealed distention with no tenderness. Bladder was barely palpable; however, suprapubic region appeared dull during percussion. Rectal examination was normal and no mass or blood was found. A foley catheter was inserted which helped decreasing the pain.

Her complete blood count was normal but serum creatinine (Cr) level was 6.2 mg/dL -it was 1.1 mg/dL before hemorrhoidectomy. Abdominopelvic ultrasonography showed free fluid in peritoneal cavity with no hydronephrosis. Considering the possibility of intestinal damage, abdominopelvic CT with contrast enema was ordered by general surgeon which revealed no bowel perforation.

**Fig. 1:**
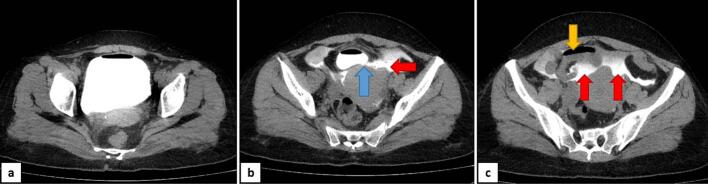
CT cystography a. the bladder with contrast agent b. bladder wall defect (blue arrow) and leakage of contrast (red arrows) c. small bowel loop (yellow arrow) and intraperitoneal contrast leakage (red arrows).

Considering obstructive urinary symptoms after her recent surgery, suprapubic dullness, free abdominal fluid, and recent increase in Cr levels, possibility of bladder retention with ensuing perforation was highlighted. To confirm the diagnosis, a CT cystography was performed revealing a bladder wall defect with the leakage of contrast into intraperitoneal bladder rupture hence confirming our suspicion i.e., SRUB into peritoneal cavity (Fig. 1). Therefore, the patient was sent to operation room for an emergent bladder surgery through suprapubic incision, repairing the bladder wall and irrigating peritoneal cavity. Serum Cr level dropped to 1.0 on postoperative day 3, so we discharged the patient with the foley catheter. 2 weeks after the surgery, a postoperative cystography was taken which showed no leakage, thus the foley catheter was removed. On her next follow-up visit she had no problem in her urination nor in defecation. This case report has been reported in line with the SCARE criteria [7].

## 3 Discussion

This case report introduces a middle-aged woman who experienced abdominal pain and obstructive lower urinary tract symptoms following hemorrhoidectomy concomitant with rise in serum Cr levels, which turned out to be SRUB into intraperitoneal cavity subsequently treated by surgical bladder repair. The aim of this case report is to underscore the importance of early recognition and diagnosis of SRUB in patients presenting with urinary retention and abdominal discomfort, particularly in the context of recent pelvic surgeries.

Spontaneous rupture of the urinary bladder (SRUB) is an uncommon and serious complication that can arise from acute urinary retention (AUR), even in the absence of trauma or malignancy [2]. Urinary retention is a known complication of anorectal surgeries, such as hemorrhoidectomy, with an incidence ranging from 15 % to 35 % [6]. The underlying mechanism for this retention is suggested to be iatrogenic insult to the nerve fibers in pelvic area leading to dysfunction in neural reflexes causing urinary retention [6]. Such bladder retention compromises the detrusor muscle's contractility and may increase intra-vesical pressure. This cascade of events could end up in bladder rupture even by minor trauma [8].

Bladder rupture is rather a rare entity in medicine which may occur as either leakage of urine in the pelvic cavity and outside of peritoneum called “extraperitoneal bladder rupture” or urine spillage into the peritoneal cavity named “intraperitoneal bladder rupture”. Such phenomenon may result in severe complications if not identified and managed promptly [5].

SRUB, however, is a particularly scarce subcategory in this regard, occurring by definition in the absence of obvious trauma including blunt trauma and instrumentation [9]. Among the reported causes of SRUB, the most commonly occurring are alcohol intoxication, lower urinary tract obstruction, bladder tumors, pelvic radiotherapy, and neurological disorders [10]. Our patient had recently undergone hemorrhoidectomy and we assume that this surgery was the underlying cause of urinary retention leading to SRUB in her.

The symptoms of bladder rupture are nonspecific including abdominal discomfort, gross hematuria, and reduced urine output [10]; it is noteworthy that such symptoms cannot distinguish whether the rupture is intraperitoneal or extraperitoneal, making early diagnosis challenging [10]. Timely diagnosis and appropriate use of imaging techniques are essential for proper management as misdiagnosis or delayed treatment can result in serious complications, including peritonitis, sepsis, and even death [5]. Laboratory tests and ultrasonography might provide some diagnostic clues [11]. However, cystography -particularly CT cystography- is the gold standard method for detecting bladder rupture and differentiating intraperitoneal versus extraperitoneal involvement [11]. In our patient, presence of obstructive urinary symptoms and abdominal discomfort after hemorrhoidectomy combined with the preliminary ultrasonography revealing free fluid in the abdomen and increased serum Cr level in the absence of hydronephrosis prompted diagnosis of intraperitoneal bladder rupture which was then confirmed by CT cystography. This highlights the necessity of sustaining a high level of suspicion for bladder rupture in individuals with a background of urinary retention, even in the absence of trauma.

Management of bladder rupture depends on the type of the rupture [5]. While urinary catheterization is enough for extraperitoneal ones, intraperitoneal ruptures require surgical intervention to repair the defect and prevent life-threatening complications [5,12]. Such practice has been advocated by European Association of Urology (EAU) and American Urological Association (AUA) guidelines [13,14]. However, there are few studies that reported successful conservative management of intraoperative bladder rupture i.e., antibiotic therapy concomitant with urinary catheter drainage without performing surgery [10]; while such incongruence is an interesting issue for future researches, we preferred to follow current guidelines for our patient hence performing surgical repair of the bladder resulting in symptom resolution and stabilization.

There are no guidelines for follow-up of the patients with SRUB after surgery, but EAU guideline suggests performing cystography in cases of complex bladder repair before removing the catheter [13]. Therefore, we performed cystography 2 weeks after the surgery which showed complete bladder wall healing. After removing the catheter, the patient had no urinary symptoms.

Our case report highlights importance of being vigilant regarding clinical presentations of bladder rupture specifically if presented in the form of SRUB. Limitation of our study is absence of objective information regarding basic bladder function of our patient before her surgeries such as urodynamic study.

## 4 Conclusion

SRUB following hemorrhoidectomy is an extremely rare but possible complication. Clinicians must be vigilant in patients presenting with urinary symptoms, abdominal pain, and increased serum Cr level in the absence of hydronephrosis after pelvic surgeries e.g. hemorrhoidectomy as intraperitoneal SRUB might be the underlying problem, misdiagnosis of which may lead to serious complications.

## Consent

Written informed consent was obtained from the patient for publication of this case report and accompanying images. A copy of the written consent is available for review by the Editor-in-Chief of this journal on request.

## Ethical approval

The ethical approval has been exempted by our institution.

## Funding

This research did not receive any specific grant from funding agencies in the public, commercial, or not-for-profit sectors.

## Declaration of competing interest

None.
